# The burden of critical illness among adults in a Swedish region—a population-based point-prevalence study

**DOI:** 10.1186/s40001-023-01279-0

**Published:** 2023-09-07

**Authors:** Carl Otto Schell, Andreas Wellhagen, Miklós Lipcsey, Lisa Kurland, Petronella Bjurling-Sjöberg, Cecilia Stålsby Lundborg, Markus Castegren, Tim Baker

**Affiliations:** 1 Department of Global Public Health, Karolinska Institutet, Stockholm, Sweden; 2 Centre for Clinical Research Sörmland, Uppsala University, Eskilstuna, Sweden; 3 Department of Medicine, Nyköping Hospital, Sörmland Region, Nyköping, Sweden; 4 Department of Anaesthesia and Intensive Care, Nyköping Hospital, Sörmland Region, Nyköping, Sweden; 5 Department of Surgical Sciences, Anaesthesiology and Intensive Care, Uppsala University, Uppsala, Sweden; 6 Department of Surgical Sciences, Hedenstierna Laboratory, Uppsala University, Uppsala, Sweden; 7 School of Medical Sciences, Örebro University, Örebro, Sweden; 8 Department of Emergency Medicine, Örebro University, Örebro University Hospital, Örebro, Sweden; 9 Department of Public Health and Caring Sciences, Uppsala University, Uppsala, Sweden; 10 Department of Patient Safety, Region Sörmland, Eskilstuna, Sweden; 11 Perioperative Medicine and Intensive Care (PMI), Karolinska University Hospital, Stockholm, Sweden; 12 Department of Physiology and Pharmacology (FyFa), Karolinska Institutet, Stockholm, Sweden; 13 Department of Clinical Research, London School of Hygiene and Tropical Medicine, London, UK; 14 Department of Emergency Medicine, Muhimbili University of Health and Allied Sciences, Dar es Salaam, Tanzania

## Abstract

**Background:**

Patients with critical illness have a high risk of mortality. Key decision-making in the health system affecting the outcomes of critically ill patients requires epidemiological evidence, but the burden of critical illness is largely unknown. This study aimed to estimate the prevalence of critical illness in a Swedish region. Secondary objectives were to estimate the proportion of hospital inpatients who are critically ill and to describe the in-hospital location of critically ill patients.

**Methods:**

A prospective, multi-center, population-based, point-prevalence study on specific days in 2017–2018. All adult (> 18 years) in-patients, regardless of admitting specially, in all acute hospitals in Sörmland, and the patients from Sörmland who had been referred to university hospitals, were included. Patients in the operating theatres, with a psychiatric cause of admission, women in active labor and moribund patients, were excluded. All participants were examined by trained data collectors. Critical illness was defined as “a state of ill health with vital organ dysfunction, a high risk of imminent death if care is not provided and a potential for reversibility”. The presence of one or more severely deranged vital signs was used to classify critical illness. The prevalence of critical illness was calculated as the number of critically ill patients divided by the number of adults in the region.

**Results:**

A total of 1269 patients were included in the study. Median age was 74 years and 50% of patients were female. Critical illness was present in 133 patients, resulting in an adult population prevalence of critical illness per 100,000 people of 19.4 (95% CI 16.4–23.0). The proportion of patients in hospital who were critically ill was 10.5% (95% CI 8.8–12.3%). Among the critically ill, 125 [95% CI 94.0% (88.4–97.0%)] were cared for in general wards.

**Conclusions:**

The prevalence of critical illness was higher than previous, indirect estimates. One in ten hospitalized patients were critically ill, the large majority of which were cared for in general wards. This suggests a hidden burden of critical illness of potential public health, health system and hospital management significance.

**Supplementary Information:**

The online version contains supplementary material available at 10.1186/s40001-023-01279-0.

## Background

The surges of unwell patients during the COVID-19 pandemic have brought critical illness to public attention. Critical illness is a state of ill health with vital organ dysfunction, a high risk of imminent death if care is not provided and a potential for reversibility [1–3]. Critical illness is not confined to specific medical specialties or the patient’s underlying diagnosis. Its defining attributes are that the condition is *severe* and *time-sensitive,* attributes strongly associated with prognosis and prompting urgent care to improve the prognosis.

Very little is known about the burden of critical illness in terms of prevalence and incidence. Information about critical illness is rarely collected in administrative data and registries. Despite this information gap, hospitals make key resource decisions concerning critical illness, such as the number of beds in intensive care units (ICUs) and high dependency units (HDUs), staffing levels and clinical routines. Improved knowledge about the burden of critical illness would assist the tailoring of health services to patient needs and contribute to improved outcomes.

Previous attempts to estimate the burden of critical illness have important methodological limitations, notably ignoring critical illness among non-ICU populations [4, 5]. The number of ICU bed-days or ICU-admissions are poor proxies for the burden of critical illness, as they depend on available ICU-bed capacity [6]: per 100,000 people, the USA and Germany have 34 and 29 ICU beds, respectively, whereas the UK and Sweden have only seven and six, respectively [7]. It is unlikely that there is a fourfold difference in the burden of critical illness between these countries—more likely that health systems define or use ICU beds differently [5, 8–11]. Adhikari et al. estimated the global incidence of adult critical illness to be 30–45 million per year through extrapolation of the incidence of specific diagnoses in a North American ICU registry to the rest of the world [2]. This figure provides a useful starting point, but the method uses retrospective indirect data, omits all critical illness outside ICUs and due to other diagnoses, and does not account for health system diversity.

An alternative method to assess the burden is to examine all patients and use their physiological status to identify critical illness. The examination of patients’ vital signs is quick in clinical practice[12, 13] and useful for assessing illness severity. Patients with severely deranged vital signs have a high risk of short-term mortality, comparable to the population of patients admitted to ICU, and timely care has the potential to reverse their condition—i.e., they are critically ill [13–19].

The aim of this study was to estimate the prevalence of critical illness in a Swedish region using a point-prevalence examination of patients’ vital signs. Secondary aims were to estimate the proportion of hospital inpatients who are critically ill and to describe the in-hospital location of critically ill patients.

## Methods

### Design and study setting

We conducted a prospective, multi-center, population-based, point-prevalence study in the Sörmland region in the central-eastern part of Sweden. Sörmland has an adult (> 18 years ) population of 228,594 (2017) with a life-expectancy similar to the national level, but slightly lower socioeconomic status [20, 21]. Sweden has tax-funded universal health coverage that includes care in hospital, and privatized hospital care is negligible in Sörmland. The region has three district hospitals for in-patient care, with a total of 470 beds of which 12 were ICU beds at the time of the study, similar to national ICU bed capacity of 5.0 per 100,000 pre-COVID-19 [22]. For tertiary care, such as neuro- and thoracic surgery, patients are referred to university hospitals in adjacent regions. The study was part of the multi-center, multi-country Critical Illness Prevalence and Outcome Study (CRISPOS) and follows the STROBE guidelines for observational studies.

### Study population

All adult (> 18 years) hospital in-patients from the Sörmland Region were examined on three specific days in 2017–2018. This included patients in the Sörmland hospitals plus those from Sörmland who had been referred and were receiving in-hospital care in university hospitals. Patients in all somatic-care wards and those in the emergency departments waiting for transfer to in-hospital care were included. The study included only patients admitted to hospitals, as we assumed the number of critically ill patients outside of hospitals was minimal. This assumption is based on the universal access in Sweden to high-quality, pre-hospital emergency services and hospital care, so that all people with acute, reversible, life-threatening illness are expected to be admitted to hospital. People with sudden out-of-hospital death and those receiving end-of-life care in their homes or in nursing homes do not have reversible disease.

We included patients with the decision, “do not resuscitate in the event of a cardiac arrest” (DNR). Most patients with DNR decisions in Swedish hospitals receive both supportive and curative care, such as oxygen, antibiotics, emergency surgery, and some are even treated in ICU [23]—with the goal of curing the acute condition and returning the patient to their usual state-of-health—therefore, they fulfil the definition of being critically ill. Moribund patients—as identified by the attending nurse—have a non-reversible condition and were excluded, as well as patients in the operating theatres, those in psychiatric wards or with a primarily psychiatric cause of admission, and women in active labor.

### Data collection

Data collection took place on 3 days in 2017–2018 that were selected to account for seasonal variability and to be feasible for the study. Before each data collection, all data collectors received a 1-day standardized training in the study methods and all medical equipment for the examinations was quality assured by medical technicians. On each occasion, in-patients in all wards and units were examined by a team of nursing students under the supervision of experienced doctors and nurses. In the university hospitals, patients from Sörmland were identified by their personal number, and traced through postal codes, referral administrative data and ambulance transport records and the patients were examined by trained doctors and nurses. For patients who were not present in their beds when the team arrived, at least one attempt was made to locate the patient later in the same day, including in other wards and units, such as recovery rooms. Data on the main diagnosis by ICD-10 code, admitting specialty, previous surgery and DNR decisions were extracted from the medical records. Population data were retrieved from Statistics Sweden. Double data entry into a spreadsheet and discrepancy checking were performed before the study database was locked.

### Definitions

**Fig. 1:**
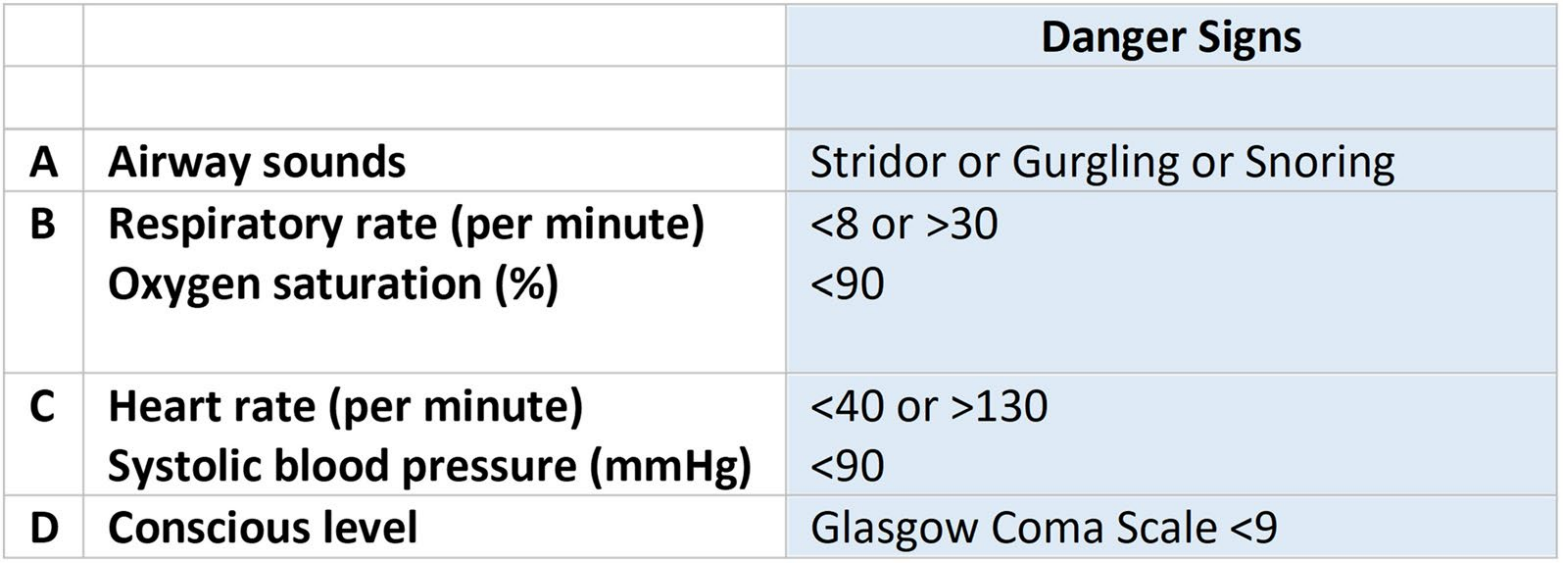
Included parameters and cutoffs for danger signs

We used the definition of critical illness as ‘*a state of ill health with vital organ dysfunction, a high risk of imminent death if care is not provided and a potential for reversibility’* [3]. The presence of one or more severely deranged vital signs was used to classify critical illness, hereafter referred to as ‘danger signs’ (Fig. 1). This method does not rely on specific diagnoses or the location of care in the hospital and is pragmatic to use in clinical practice. We used a-priori decided cutoffs for danger signs adapted from the rapid response system in Karolinska University Hospital and previous research [17, 24, 25].

The in-hospital location where the patient was being treated was defined as’ICU’ (care in a unit specified as ICU by the hospital),’HDU’ (care in a post-operative recovery area or a bed with continuous monitoring of vital signs and availability of non-invasive ventilation) or ‘general ward’. The main diagnosis was defined by using the ICD-10 codes written in the medical records.

### Data analysis

The prevalence of critical illness was calculated as all patients with one or more danger sign identified in the three data collections divided by the number of adults in the Sörmland region > 18 years in the December 31st, 2017, census [multiplied by three to match the three data collections (*N* = 228,594 × 3 = 685,782)]. For the secondary aim of estimating the proportion of hospital in-patients who were critically ill, the total number of examined patients was used as the denominator. Missing data for vital signs were classified as ‘not a danger sign’, using imputation to the most frequent value. Descriptive statistics were used for patient characteristics. In applicable analyses, confidence intervals of 95% were used. Stata /IC 15 (Stata Corp, College Station, TX) was used for the statistical analyses.

### Sensitivity analyses

As there are no generally agreed criteria for critical illness, we performed some sensitivity analyses. First, due to the argument that a DNR decision could indicate non-reversible disease, we classified all patients with DNR decision as being ‘not critically ill’. Second, due to the possibility that intensive therapies could normalize physiological parameters, we classified all patients receiving care in ICU and HDU as critically ill—irrespective of their vital signs. Third, we calculated the patients’ National Early Warning Scores (NEWS)[15] and classified those with a score of ≥ 7 or alternatively ≥ 5 and as critically ill (Additional file 1: Fig. S1).

#### Ethical considerations

The study followed the principles of the Helsinki declaration and its subsequent revisions. Ethical permission was granted by the Swedish Ethical Review Board, Stockholm (EPN 2017/1907-31/1). Participants were included after receiving written information, and as the study was low-risk and the participation of patients with reduced conscious level was vital for the validity of the study, consent was assumed from such patients, in the absence of any objection from the patients or next-of-kin.

## Results

**Fig. 2:**
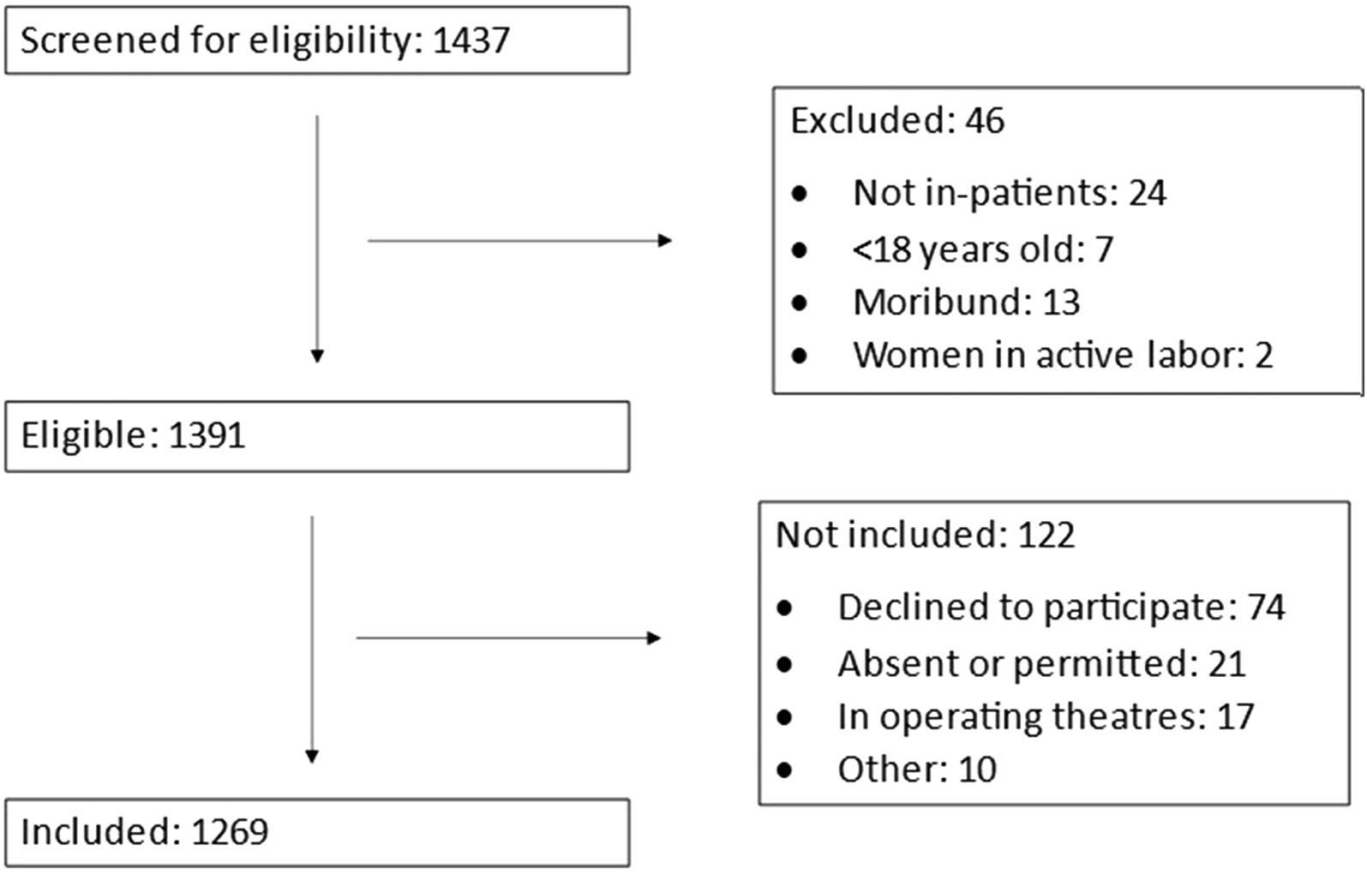
Flow diagram of the inclusion process

A total of 1437 patients were screened in the hospitals, 1391 were eligible and 1269 patients were included in the study (Fig. 2). The patients’ median age was 74 years (IQR 62–83) and 640 (50%) were female. Of all patients, 820 (65%) were cared for in the departments of internal medicine, and the remainder in the surgical, and obstetrics and gynecology departments (Table 1). There was a large variety of diagnoses (*n* = 822), and 145 patients (11%) had one of the three most common diagnoses, (pneumonia, stroke, or heart failure). During their time in hospital before inclusion in the study, 208 (16%) of patients had undergone a surgical procedure and 233 (18%) had a DNR decision made for their care. There were 1209 (95%) patients who received care in general wards, 32 (3%) in HDUs and 29 (2%) in ICUs. Of all patients, 47 (4%) had been transferred for care in university hospitals.

**Table 1:** Characteristics and care of the included patients by critical illness

	All *N* = 1269	Critically ill *n* = 133	Non-critically ill *n* = 1136	
Age median (IQR)	74 (62–83)	76 (69–87)	73 (71–82)	*p* < 0.01
Sex				
Female	640 (50%)	59 (44%)	581 (51%)	
Male	629 (50%)	74 (56%)	555 (49%)	*p* = 0.14
Specialty				
Medicine	820 (65%)	115 (86%)	705 (62%)	
Surgery	386 (30%)	18 (14%)	368 (32%)	
Obstetrics and Gynecology	63 (5%)	0	63 (6%)	*p* < 0.01
Had surgery in hospital				
No	1061(84%)	125 (94%)	936 (82%)	
Yes	208 (16%)	8 (6%)	200 (18%)	*p* < 0.01
Location in hospital				
Ward	1208 (95%)	125 (94%)	1083 (95%)	
HDU	32 (3%)	3 (2%)	29 (3%)	
ICU	29 (2%)	5 (4%)	24 (2%)	*p* = 0.48
Hospital level				
District hospitals	1222 (96%)	127 (95%)	1095 (96%)	
University hospitals	47 (4%)	6 (5%)	41 (4%)	*p* = 0.60
End of life decision				
Do resuscitate	1036 (82%)	74 (56%)	962 (85%)	
Do not resuscitate (DNR)	233 (18%)	59 (44%)	174 (15%)	*p* < 0.01

Not all percentages add up to 100% due to rounding. HDU: High Dependency Unit; ICU: Intensive Care Unit

**Fig. 3:**
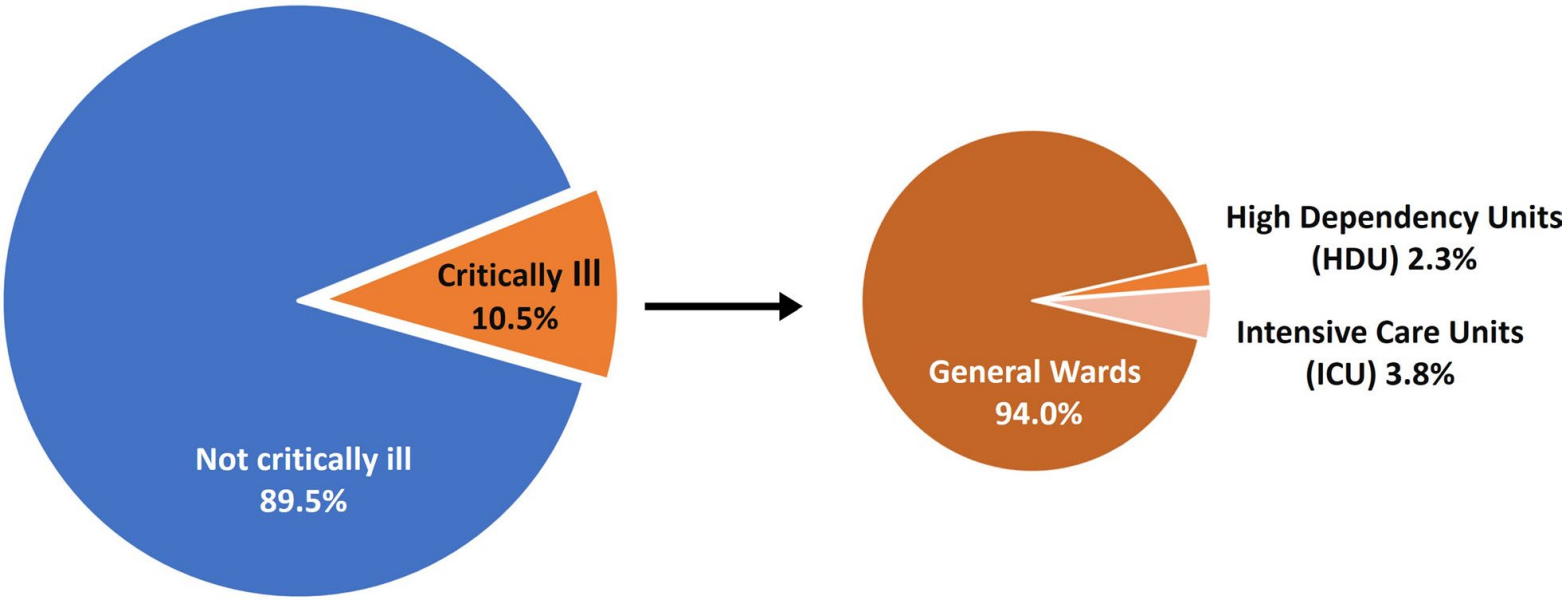
Proportion of critically ill patients among all hospital in-patients and the in-hospital location of critically ill patients

Critical illness was present in 133 patients. This gave an adult population prevalence of critical illness per 100,000 people of 19.4 (16.4–23.0). The proportion of patients in hospital who were critically ill was 10.5% (8.8–12.3) (Fig. 3). Among the critically ill, 125 [94.0 (88.4–97.0)] were cared for in general wards, 3 [2.3% (0.7–7.7)] in HDUs and 5 [3.8% (1.6–8.7)] in ICUs (Fig. 3).

Out of the 133 critically ill patients, 74 [55.6% (47.2–64.1)] were male, 8 [6.0% (2.0–10.1)] had undergone surgery and 115 [86.5% (80.1–92.3)] were admitted to medical departments. Fifty-nine [44.3% (36.8–52.8)] had a DNR decision (Table 1). Out of the 10,526 vital signs assessments conducted, there were 25 missing values (0.2%).

In the sensitivity analyses, the prevalence of critical illness per 100,000 people was 10.8 (8.5–13.6) when excluding patients with a DNR decision; 27.1 (23.5–31.3) when also including all patients in ICUs and HDUs as critically ill; 11.1 (8.9–13.9) using NEWS ≥ 7 to classify critical illness and 28.4 (24.7–32.7) using NEWS ≥ 5 to classify critical illness. (Additional file 1: Tables S2 and S3).

## Discussion

We have found a population prevalence of adult critical illness per 100,000 people of 19.4 in the Sörmland Region of Sweden. Of all hospitalized patients, 10.5% were critically ill and of these critically ill, 94% were cared for in general wards. To our knowledge, this is the first attempt to estimate the burden of critical illness at population level using prospective enrolment and examination of patients.

### The prevalence of critical illness

This burden of critical illness is higher than previously considered, but comparisons with previous estimates are challenging. Since the burden of acute illnesses is often measured using the *incidence*, (the flow of cases into the system per time unit), a conversion of our prevalence estimate is needed. Using an assumption that critical illness on average has a duration of 1.1 days (based on the proxy of the median duration of patients’ care in Swedish ICUs) [26], our findings would correspond to 17.6 new cases of critical illness per 100,000 people per day. This number is substantially higher than the estimate by Adhikari et al. of 1.3–1.9 per 100,000 people per day in Europe [2]. The difference could be explained by our more liberal criteria for critical illness rather than limiting inclusion to patients with certain syndromes and those treated in ICUs. We do not believe that our criteria were too liberal. Severely abnormal vital signs have been shown to identify a group of patients with a high risk of death—indeed their risk of 26–28% 30-day mortality [13] is higher even than the risk for patients admitted to ICUs (17%) [19]. The burden of critical illness in our study is also larger than the burden of major acute diagnoses that receive substantial public, research, and policy interest. There are 0.5 strokes [27], 2.7 hip fractures [28], and 2.8 [29] myocardial infarctions in Sweden per 100,000 people per day, with 30-day mortalities of 11% [27], 8% [30], and 8% [31], respectively. Critical illness appears to be a larger health issue than is usually recognized.

There are important policy implications of this large burden of critical illness. The health system requirements need to be specified for these patients, as do the competencies of health workers to care for them, and the clinical systems and routines to prioritize [32, 33] for them to be quickly *identified* as critically ill and promptly *treated*—even when resources are limited such as at 3 am on a Sunday night [34]. Re-designing health services to have an increased focus on critical illness, in the same way as the longstanding focus on diagnoses with high mortalities [35–37], would target the highest risk patients and—as they are common—could be favorable for quality improvement, research, and innovation. Improving care of severely ill patients would benefit patients suffering from any underlying condition and have the advantage of improving care for patients lacking a definitive diagnosis, those with multimorbidity and those who have been mis-diagnosed.

### The proportion of patients in hospital that are critically ill

Among all in-patients, 10.5% were critically ill, a finding in-line with previous research. Studies from university hospitals in Finland and Sweden report proportions of patients with single parameter signs of 8.4% and 12% and NEWS ≥ 7 of 6.0% and 6.5%, respectively [13, 38]. In our population-based study, most patients were treated in district hospitals, and of these patients, 10% were critically ill (versus 13% in the university hospitals). While it may be thought that most patients with critical illness would be referred to ICUs and to larger hospitals, the findings do not support that perception.

### The in-hospital location of critically ill patients

A large majority of critically ill patients (94%) were cared for in general wards. There are, to our knowledge, no previous studies of the in-hospital location of critically ill patients, but the presence of very ill patients in general wards and not just in HDUs and ICUs has been described in work on sepsis, early warning systems (EWS), rapid response teams (RRT)—and from low-resource settings [16, 39–44]. While critical illness does not equal need of care in an ICU, the implications of this finding are debatable [45]. An argument could be proposed that an ICU-bed capacity corresponding to that in the USA and Germany (> 20 per 100,000 people) could be needed to care for these patients. However, from a health system perspective, it would be more rational to provide care at the lowest effective level to maximize the use of resources [46, 47]. ICUs may offer better care in some cases [48], but at a high cost [49]—and many countries in the world have less than one ICU bed per 100,000 people [50]. ICU care did not provide better outcomes for patients lacking a strong ICU indication and was not always cost-effective in the COVID-pandemic [47, 51]. For a large proportion of the critically ill, HDUs providing less advanced critical care together with optimized care of critical illness in general wards may be a better approach to prevent deterioration, the need for ICU care and death [47, 52]. This requires that health systems ensure adequate provision of fundamental critical care in the general wards, such as through training of staff, the use of EWS and RRT, and focused quality improvement [52–54]. A recent consensus has specified ‘Essential Emergency and Critical Care’, 40 clinical processes that are effective, lifesaving and feasible to deliver in general wards and all other parts of hospitals [34].

### Strengths and limitations

The strengths of the study include the use of criteria to define critical illness that are neither dependent on the underlying diagnosis nor the hospital location; the prospective examination of all study participants by trained data collectors and quality secured equipment; the few missing data points; and the high inclusion rate of patients from all types of wards and hospitals in which a well-defined population are admitted for care, including referrals to high level care in other regions.

The study had limitations. First, the pragmatic vital signs-based criteria used may have missed some high-risk patients whose vital signs were insufficiently deranged or had been stabilized through care. Conversely, some patients with adapted physiology due to chronic disease may have been classified as being critically ill. Second, for logistical reasons, we could neither include patients in the operating theatres nor patients in the emergency units awaiting a decision about hospital admission, some of whom may have been critically ill. Third, data collections took place before the COVID-19 pandemic, in the daytime on weekdays and the prevalence may differ over time and between weekends and nights [55]. Fourth, the prevalence estimates changed when alternative criteria for critical illness were used in the sensitivity analyses, highlighting the challenge of identifying critical illness and the high importance of a process towards agreed criteria for critical illness [3]. Fifth, we assumed that there is no critical illness in the community. If this assumption is false, the burden of critical illness may be greater than our estimate. Finally, this study was from one region in one country. However, we have no reason to believe that there are substantial differences between settings similar to Sweden, and a cautious transfer of the findings could be useful for many health systems.

## Conclusion

In this prospective, multi-center population-based study, we have found a prevalence of adult hospital-treated critical illness of 19.4 per 100,000 people. Among hospitalized patients, one in ten were critically ill, a large majority of which (94%) were cared for outside of ICUs and HDUs. Our estimates are higher than previous, indirect estimates, suggesting a hidden burden of critical illness of potential public health, health-system, and hospital management significance.

### Supplementary Information

**Additional file 1: Figure S1.** Parameters for National Early Warning score (NEWS).**Table S2.** Sensitivity analyses. Prevalence of adult hospital-treated critical illness and the proportion of critically ill patients in hospital.**Table S3.** Sensitivity analyses. In-hospital location.

## Data Availability

Access to deidentified data may be provided to researchers after provision of a study protocol and justification to the corresponding author.

## Abbreviations

DNR: Do not resuscitate in the event of a cardiac arrest
EWS: Early warning systems
HDU: High dependency unit
ICD-10: International Classification of Diseases, Tenth Revision
ICU: Intensive care unit
NEWS: National Early Warning Score
RRT: Rapid response team
