# Supplementary material for: The burden of critical illness among adults in a Swedish region—a population-based point-prevalence study
Source: Eur J Med Res. 2023 Sep 7;28:322. doi: 10.1186/s40001-023-01279-0 (PMC10483802; doi:10.1186/s40001-023-01279-0)
Supplement: Supplementary file 1 — Additional file 1: Figure S1. Parameters for National Early Warning score (NEWS).Table S2. Sensitivity analyses. Prevalence of adult hospital-treated critical illness and the proportion of critically ill patients in hospital.Table S3. Sensitivity analyses. In-hospital location. [file 40001_2023_1279_MOESM1_ESM.docx]

# ADDITIONAL FILE

The burden of critical illness among adults in a Swedish region – a population-based point-prevalence study.

***Figure S1:*** *Parameters for National Early Warning score (NEWS)*

***Table S2:*** *Sensitivity analyses. Prevalence of adult hospital-treated critical illness and the proportion of critically ill patients in hospital*

***Table S3:*** *Sensitivity analyses. In-hospital location.*

***Figure S1:*** *Parameters for National Early Warning score (NEWS)*

|  |  | **National Early Warning Score (NEWS)** | | | | | | |
| --- | --- | --- | --- | --- | --- | --- | --- | --- |
|  |  | 3 | 2 | 1 | 0 | 1 | 2 | 3 |
|  |  |  |  |  |  |  |  |  |
| **B** | **Respiratory rate (per minute)**  **Oxygen saturation (%)**  **Inspired oxygen** | <9  >92 | 92-93  Yes | 9-11  94-95 | 12-20  >95  No |  | 21-24 | >24 |
| **C** | **Heart rate (per minute)**  **Systolic blood pressure (mmHg)** | <41  <91 | 91-100 | 41-50  101-110 | 51-90  111-219 | 91-110 | 111-130 | >130  >219 |
| **D** | **Conscious level** |  |  |  | A |  |  | V or P or U |
| **E** | **Temperature (°C)** | <35.1 |  | 35.1-36.0 | 36.1-38.0 | 38.1-39.0 | >39.0 |  |

***Table S2:*** *Sensitivity analyses. Prevalence of adult hospital-treated critical illness and the proportion of critically ill patients in hospital*

|  | Prevalence per 100,000 people (95% CI) | Proportion critically ill among hospital in-patients (95% CI) |
| --- | --- | --- |
|  |  |  |
| Critical illness  n=133 | 19.4  (16.4-23.0) | 10.5%  (8.9-12.3) |
|  |  |  |
| Critical illness excluding DNR  n=74 | 10.8  (8.6-13.5) | 5.8%  (4.6-7.3) |
| Critical illness + all in ICU & HDU  n=186 | 27.1  (23.5-31.3) | 14.7%  (12.8-16.7) |
| NEWS ≥7  n=76 | 11.1  (8.9-13.9) | 6.0%  (4.7-7.4) |
| NEWS ≥5  n=195 | 28.4  (24.7-32.7) | 15.4%  (13.4-17.5) |

***Table S3:*** *Sensitivity analyses. In-hospital location.*

|  | In hospital location of the critically ill patients | | |
| --- | --- | --- | --- |
|  | **Ward** | **HDU** | **ICU** |
| Critical illness  n=133 | 125 (94%) | 3 (2%) | 5 (4%) |
|  |  |  |  |
| Critical illness excluding DNR  n=74 | 68 (92%) | 2(3%) | 4(5%) |
| Critical illness + all in ICU & HDU  n=186 | 125(67%) | 32(17%) | 29(16%) |
| NEWS ≥7  n=76 | 70 (92%) | 3(4%) | 3(4%) |
| NEWS ≥5  n=195 | 174(89%) | 6(3%) | 15(8%) |
